# Effect of Embedded Thin-Plies on the Charpy Impact Properties of CFRP Composites

**DOI:** 10.3390/polym14091929

**Published:** 2022-05-09

**Authors:** Hassan Alshahrani, Tamer A. Sebaey

**Affiliations:** 1 Department of Mechanical Engineering, College of Engineering, Najran University, Najran 11001, Saudi Arabia; 2 Engineering Management Department, College of Engineering, Prince Sultan University, Riyadh 12435, Saudi Arabia; tsebaey@psu.edu.sa; 3 Mechanical Design and Production Department, Faculty of Engineering, Zagazig University, Zagazig 44519, Sharkia, Egypt

**Keywords:** thin ply laminates, CFRP composites, Charpy, impact energy, finite element analysis (FEA)

## Abstract

In this study, different configurations of epoxy composite laminates that contained thin plies were prepared and characterised for sudden impact load bearing applications. The primary aim of this investigation was to develop a hybrid epoxy-based thin ply composite for aerospace and automotive applications that would be tolerant of high impacts. The impact properties of the selected configurations were investigated both experimentally and numerically under low-velocity Charpy impact loading conditions. Furthermore, any damage to the laminates was evaluated with an emphasis on the identification of dominant damage mechanisms and locations. This included a comparison between the laminates that were made from traditional plies and the thin ply laminates in terms of their absorbed energy and failure modes. The results revealed that the integration of thin plies into normal ply had a major effect on the amount of absorbed energy under flatwise conditions: up to 8.7 J at a cut-off angle of 90°. However, edgewise conditions produced a maximum observed energy of 10.0 J for the thin plies that were surrounded by normal plies (Plate 3). The damage assessments showed the increased damage resistance of the hybrid thin ply composites due to their uniform stress distribution. The traditional ply composites incurred large deformations from the impact loads. Moreover, it was noted that delamination formed in the middle regions of the traditional plies. The FEM model analysis revealed that it was capable of accurately predicting the absorbed energy for different configurations of composites, which were prepared and analysed experimentally. Both the experimental and numerical values were very similar to each other. These impact damage assessments improved the thin ply composites so that they could be used as working materials for applications that are prone to high loads, such as the aerospace, defence, automotive and structural industries.

## 1. Introduction

Polymer matrix composite laminates are widely used in several sectors, such as the aerospace industry, piping and marine structures. In contrast to traditional metallic structures, the key advantages of composite laminates are their high specific stiffness, strength and dimensional stability [1]. In addition to the strength and stiffness, these composites also possess good fatigue, creep and sudden load bearing properties. The presence of fibres and their orientation are the factors that are responsible for improving these parameters in the composites. Thus, many researchers around the world are now focusing their research on the development of polymer matrix composites that use large plies [2]. With the increasing advancements in this field, thin ply composites have opened doors to alternative possibilities for structural design. These composites have cured ply thicknesses of less than 0.0025 in., while commercially available pre-pregs currently have ply thicknesses as low as 0.00075 in. A composite with a standard ply thickness, for comparison, would have a cured ply thickness of 0.0055 in. or larger. Due to their unique structural characteristics, such as improved damage tolerance, resistance to microcracking, improved ageing and fatigue resistance, reduced minimum-gage thickness and increased scalability, thin ply composites have the potential to reduce structural mass while increasing performance [3]. Thin ply composites can be used for a variety of applications in both aeronautics and space engineering because of these features. Preliminary studies have demonstrated that a mixture of thin and regular ply layers can boost the notched tensile strength of composite laminates by 30%. As a result, the selective use of thin plies in composite aircraft constructions could drastically reduce their bulk. Space applications also include a wide range of possibilities. Thin ply composites are an excellent candidate for deep-space habitation structures within which hermeticity is critical due to the resistance of the composites to microcracking and fatigue [4].

These composites have created additional space and freedom for ply orientations, thereby increasing the design possibilities for composite components. These composites have been extremely useful in the design of thin structures that would otherwise be restricted in terms of the number of plies and their equivalent orientations. The use of thin ply composites can increase damage tolerance and strength while reducing ply thickness. By using thin ply CFRP and glass fibre pre-pregs [5] or metals [6,7], more design possibilities for material–layered hybrids can be achieved. Comprehensive review articles on thin ply composite laminates and thin ply polymer composite materials have been recently published by Galos [8] and Arteiro et al. [9], respectively.

The residual strength of laminates can be greatly affected by impact damage, the development of local cracks and delamination [10]. Some of the shortcomings of laminated CFRP composites in aerospace applications include damage tolerance and impact resistance, as any visible impact damage can have a noticeable and significant effect on the residual mechanical properties of the structure. The damage tolerance and impact resistance of composite laminates are determined based on several elements. These elements include resin toughness, reinforcement architecture, impactor geometry, stacking sequence, environmental circumstances and fibre/matrix hybridisation [11]. The Charpy impact test is a basic mechanical test that is used to evaluate the toughness of materials and determine the crack fracture toughness of materials. The simulation and optimisation of the Charpy impact test can simplify experimental processes and improve experimental accuracy [12]. The ability of a material to absorb energy during elastic deformation, plastic deformation and fracture is referred to as crack impact toughness. The energy that is absorbed by a material fracture during the Charpy impact test is about equal to the potential energy that is lost at the starting position of the pendulum and the highest point after the material is fractured. Only the moment when the pendulum strikes the material is examined during computer simulations and finite element analysis is then utilised to calculate the toughness of the material. Yokozeki et al. [13] compared the impact properties of laminates that were manufactured from thin ply pre-pregs to those made from standard pre-pregs. Their results showed that the use of thin ply pre-pregs leads to improvements in damage resistance properties against matrix cracking and delamination. Shin et al. [14] reported a 10% increase in the strength of thin ply laminates. Yokozeki et al. [15] investigated the damage characteristics of carbon fibre/toughened epoxy thin ply and conventional laminates that were subjected to transverse loadings. Their results demonstrated a clear difference between the damage accumulation processes of conventional laminates and thin ply laminates. The effects of ply thickness on the impact damage mechanisms in CFRP laminates were investigated by Saito et al. [16] and it was found that thin ply laminates show 23% higher strength than standard ply laminates.

Arteiro et al. [17] conducted research using different analysis methods, including inherent flaw, linear elastic fracture mechanics, finite fracture mechanics, average stress and point stress, to predict the damage capability of non-crimp fabric–thin ply laminates. On the other hand, Sebaey et al. [18] considered the low-velocity impact resistance of hybrid laminates that consisted of both thin and normal plies (80 gsm and 300 gsm). The researchers showed that there is a 15% rise in the delamination threshold load of thin laminates in comparison to conventional laminates. In addition, they concluded that there is also a 15% increase in the “CAI” strength of thin ply laminates. Sasikumar et al. [19] confirmed that there are positive effects of ply-level hybridisation laminates. Their study demonstrated that thin ply laminates undergo more fibre damage compared to laminates that have thick plies. As a result, there is a significant reduction in the “CAI” strength of thin ply laminates. Moreover, the “CAI” strength of a hybrid laminate that consists of 0° plies and thin plies is as much as 40% higher compared to laminates that only contain thin plies.

Soto et al. [20] worked on numerical simulations of the CAI strength and low-velocity impact of thin ply fabric laminates. The results of their numerical simulations showed the importance of the delamination damage that occurs on several interfaces. The study also showed that delamination is propagated after damage initiation until the final stage is attained, which is primarily regulated by fibre failure. Droździel et al. [21] investigated fibre metal laminates that contained a thin ply carbon fibre reinforced polymer and aluminium layers under low-velocity impact conditions. The impact energies ranged from 2.5 J to 30 J. The findings showed that the metal layers play an important role in terms of protection. However, the authors also suggested that further investigations into the effects of thin ply are required. Caminero et al. [22] conducted a characterisation and evaluation of the damage to CFRP composite laminates using different stacking sequences that were subjected to low-velocity impacts and flexural loading. They found that laminates with ±45 angle ply exhibit the best impact performance in terms of averaged absorbed energy. The transition between damage modes in thin and ultra-thin ply laminates under out-of-plane loading was studied by Wagih et al. [23]. Their outcomes showed that fibre breakage appears earlier in ultra-thin ply laminates. The process of damage evolution in thin ply laminates was investigated by Naderi and Iyyer [24] using a micromechanical approach and three-dimensional finite element analysis. Their outcomes showed that fibre–matrix interfacial failure is the dominant failure mechanism for the subsequent initiation and propagation of cracks. Zachariah et al. [25] studied the application of hybrid aramid and carbon fibre reinforced polymer thin ply laminates during enhanced static and dynamic transverse loading behaviours. Based on their report, laminates with two layers of aramid fabric at a (0/90) and (±45) orientation on their outer surface display a better impact behaviour in terms of average impact strength and absorbed energy.

Several recent studies have been published on the usage of thin plies and they have concluded that the usage of thin ply pre-pregs improves damage resistance properties in terms of delamination and matrix cracking. However, fibre breakage is one of the most common damage mechanisms in laminates with thin plies. Moreover, Sebaey et al. [26] reported that fibre breakage damage modes still need further investigation with impact energies of greater than 45 J. Therefore, the degrees of fibre breakage should be investigated at high levels of impact energy using the Charpy test. Similar to obtaining high-strength thin plies for aerospace applications, encapsulation materials also hold just as much importance as the fibres. Epoxy resins are one of the forms of material that are high strength, resistant to corrosion and electrically insulative that have a wide scope in high-performance composite applications [27]. Usually, two-component epoxy resins are very brittle and have negligible elongation. However, when reinforced with high-toughness fibre, the strength of these resins is superior to any other class of resins, such as vinyl or polyester. The chemical and abrasion resistance of epoxy resins are also higher than other resins; thus, epoxy-based thin ply composites are highly recommended in many engineering applications [28].

To the best of the authors’ knowledge, there are no available studies on the influence of embedded thin plies in CFRP composite laminates using epoxy resin in terms of high-impact energy under Charpy test conditions. Since the stability of thin plies is better than that of thick plies, an evaluation using the Charpy impact test condition was the most suitable for this study [29]. Moreover, as thin plies are bonded together more uniformly, their load sharing ability and crack suppression phenomena are higher than those of other models [30]. Since composites are mainly deployed in structural, automotive and aerospace structures, the study of the impact toughness provides more powerful decision-making results before the implementation of these composites in practical applications. Thus, the current research examined the effects of the impact resistance of embedded thin plies on the Charpy impact properties of CFRP composites, both experimentally and numerically. This included a comparison between those made of traditional composite layers at different cut-off angles. The dominant damage mechanisms and locations for both thin ply laminates and laminates that were made from traditional plies were also studied and compared using numerical analysis.

## 2. Materials and Method

### 2.1. Materials and Sample Preparation

**Figure 1:**
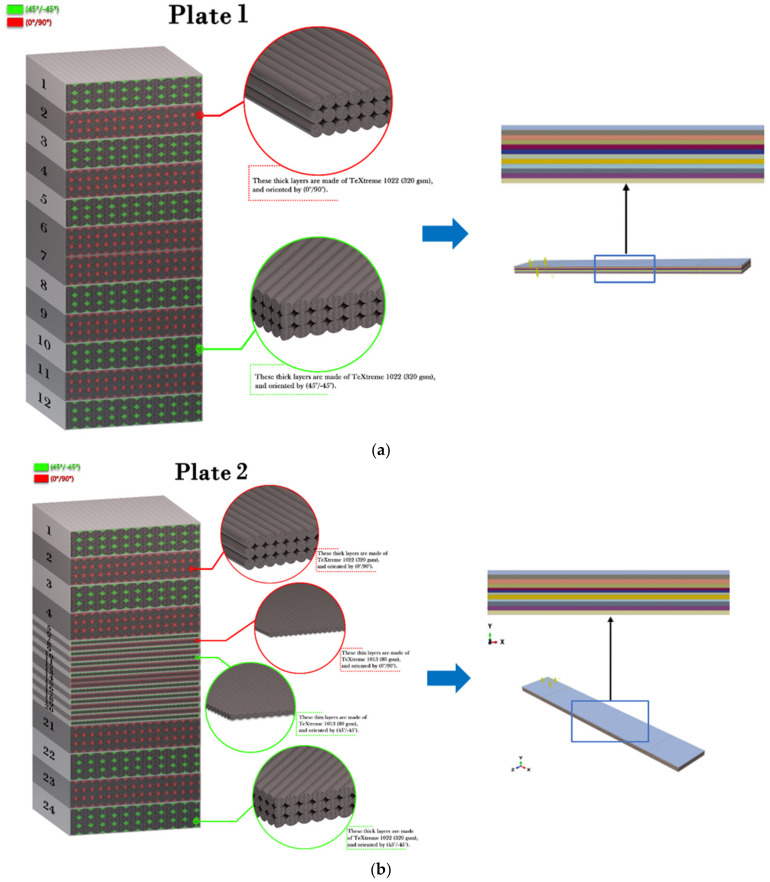

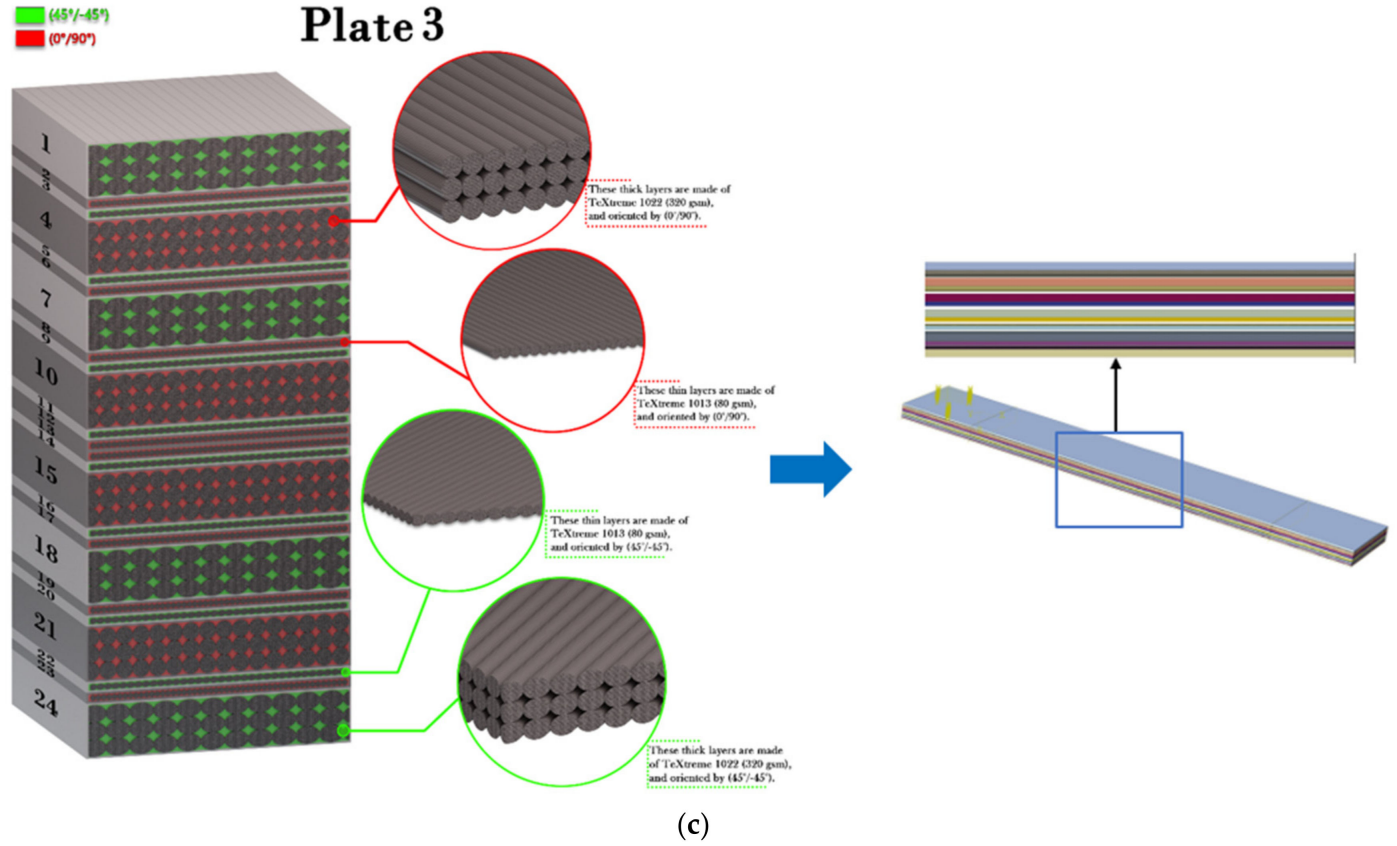
Cross-sections of the three different groups of samples and their stacking schemes; (**a**) baseline (reference) laminate of traditional plies and its model, (**b**) hybrid (standard and thin plies) laminate A and its model, (**c**) hybrid (standard and thin plies) laminate B and its model.

**Figure 2:**
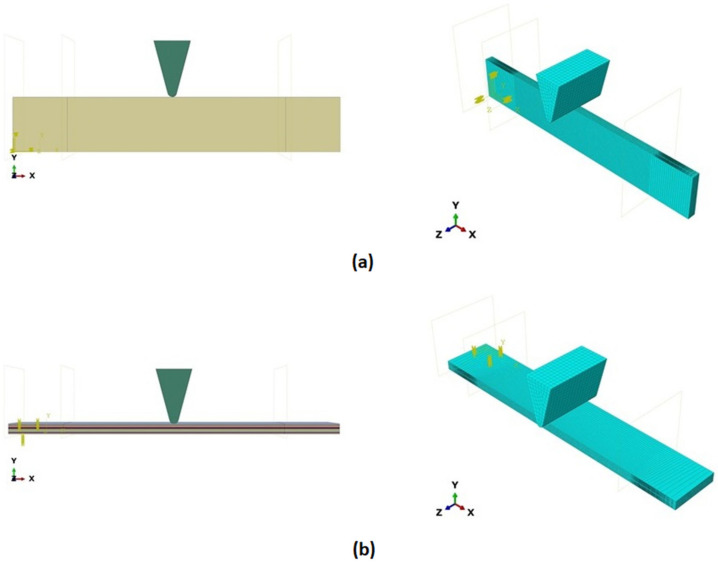
Model of the Charpy impact test; (**a**) edgewise impact direction, (**b**) flatwise impact direction.

**Figure 3:**
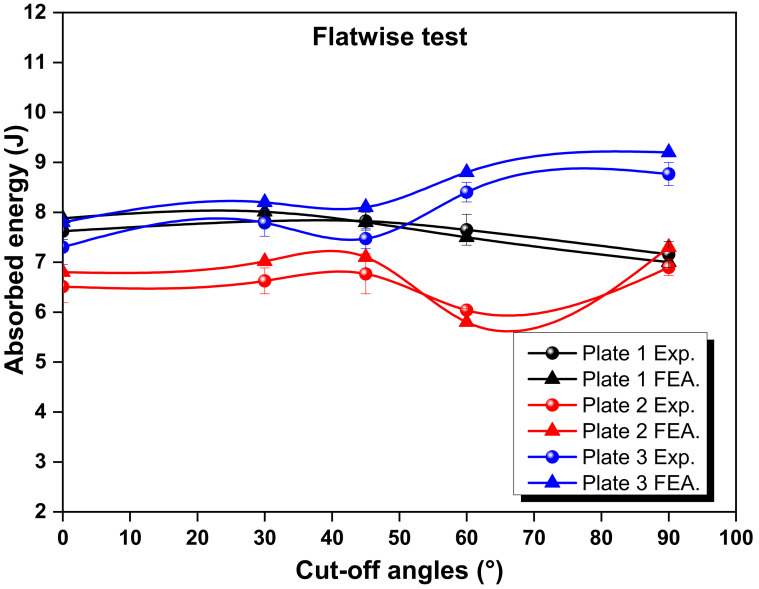
Absorbed energy at various cut-off angles for the flatwise test vs. finite element modelling.

Two different woven carbon fibre fabric grades were selected for this study. The thin plies of fabric–epoxy composites were provided by Oxeon. The ply thickness of the thin plies was 0.085 mm compared to 0.33 mm for typical ply. An epoxy resin that was supplied by SHD composites, which was known as MTFA500 (DF044), was used as the matrix to prepare the samples using three different stacking sequences. The composites that were made using epoxy had very high adhesion strength. The wavy nature of the thin plies and surrounding plies offered mechanical locking with the resin layer, which was likely to resist sliding and peeling off [31,32]. Thus, no peel-off conditions were assumed. These three different groups of selected samples were manufactured as follows: (1) the baseline (reference) laminate consisted of 12 layers of traditional plies, had a thickness of 3.98_±0.039_ mm (see Figure 1a); (2) the hybrid laminate A was manufactured from both standard and thin plies and had a thickness of 4.10_±0.027_ mm and the thin plies were located at the mid-plane of the laminate (see Figure 1b); (3) the hybrid laminate B was manufactured from both standard and thin plies, had a thickness of 4.11_±0.033_ mm and two thin plies surrounded each traditional ply (see Figure 1c). The three different groups of selected samples and their stacking sequences are presented in Figure 1. After this stage, the samples were cut as per the Charpy impact test. Different cut-off angles (0°, 30°, 45°, 60° and 90°) with respect to impact direction were also investigated for all groups of selected samples. The mechanical properties of the CF–epoxy composites are presented in Table 1, which were measured according to the ASTM-D3039 standard.

### 2.2. Charpy Impact Test

Compared to other low-velocity impact tests, Charpy tests can generate higher energy impacts and more damage on smaller surface areas. This test is capable of creating useful comparison data in a simple and efficient manner. To evaluate the impact damage resistance of embedded thin ply laminates, a motorised pendulum impact Charpy test was utilised. All of the Charpy experiments were conducted with a pendulum assembly that had an effective weight of 54.4 kg and a length of 762 mm, which resulted in an impact velocity of 5.28 m/s, as well as impact energy of 750 J. The Charpy striker that was attached to the pendulum head was an 8-mm striker with a weight of 950 g. The samples were all 60 mm long and 10 mm wide and they were all supported by anvils with a 40-mm spread. Except for the specimen sizes, which were chosen based on the machine capacity in terms of length and the recommended specimen size in the literature, the approach that was followed was the same as ASTM-D6110-10. It should be noted that before each series of testing, the friction angle had to be correctly calibrated and checked. This meant that when a free swing was performed, the energy absorbed field should have been zero. A complete swing of the pendulum from the locked position with no in situ specimen is referred to as a free swing. Both edgewise and flatwise tests were conducted for each sample configuration. In addition, three test specimens were used for each test. This resulted in a test matrix of three plates, five cut-off angles and three test specimens per condition, which totalled 45 tests.

### 2.3. Finite Element Analysis

During the Charpy impact tests, the commercial finite element program ABAQUS CAE 2020 was used to predict the absorbed energy and failure modes of all of the specimens with the different configurations of composite laminates at various cut-off angles (0°, 30°, 45°, 60° and 90°). The explicit dynamics model was used to carry out the simulation work. According to the ASTM-D6110-10 testing conditions, the geometrical Charpy impact testing model was created, as shown in Figure 2. A graded mesh was created, for which the regions at the impact points in the edgewise and flatwise directions on the thickness and width of the plates, respectively, were more finely meshed. In total, 12 layers of C3D8R modelled the three plates or laminates: an 8-node linear brick, reduced integration and hourglass control. The striker was defined as a rigid body that was relative to the plate, which exhibited normal and tangential behaviour during surface-to-surface contact interactions. The number of elements in the striker was 1350. Dynamic and explicit steps were defined using constant times. The element size was 0.1 mm, which was selected based on the convergence test. The impact velocity was set at 5.28 m/s in 0.2 ms.

For the Charpy impact analysis, a 3D model was created using the solid module and was defined using the Abaqus CAE explicit dynamic model. The Charpy test samples had 243,000 elements. The smaller the mesh element, the more accurate the calculation result, but the calculation amount also increased. As the force was concentrated on the fracture area during the Charpy impact tests, the sample areas could be split into fracture and non-fracture areas when dividing the grid and the unit grid size could be reduced within the fracture area to improve data accuracy. The computational complexity of the simulation runs could be reduced by increasing the unit grid size within the non-fractured areas. At the same time, the grid was divided into rectangular grids as much as possible, taking into account the transfer characteristics of the force field and the deformation that was produced during the investigation. The model was expanded based on the two-dimensional section in this study and 3D mesh was chosen as the mesh for the experimental fracture model. The computer simulations were considered at the moment of impact. For this purpose, potential energy was converted into kinetic energy at the moment of impact by considering the impact velocity.

To develop a model that could simulate the potential damage modes of three different plates, cohesive elements were created at the interface between the two adjacent plies. The elastic properties of the interface material were defined using traction–separation behaviour, as shown in Table 2. The quadratic traction–interaction failure criterion was chosen for damage initiation within the cohesive elements. In addition, a mixed-mode energy-based damage evolution law that was based on the Benzeggagh–Kenane (BK) criterion was used for damage propagation. Finally, the primary models were examined, the damage parameter situations were assessed and the final results were compared to the experimental findings.

## 3. Results and Discussion

### 3.1. Absorbed Energy

Three tests were conducted for each condition to ensure the repeatability of the impact results. The absorbed energy results were averaged across the three tests. Different configurations were considered for the analysis of absorbed energy. To investigate the energy absorption of the different plates, a series of Charpy impact tests was performed considering different cut-off angles (0°, 30°, 45°, 60° and 90°) with respect to the impact direction. The tests were conducted for a flatwise impact, in which the impact load was applied in the out-plane direction of the laminate, and an edgewise impact, in which the impact load was applied in the in-plane direction (see Figure 2).

**Figure 4:**
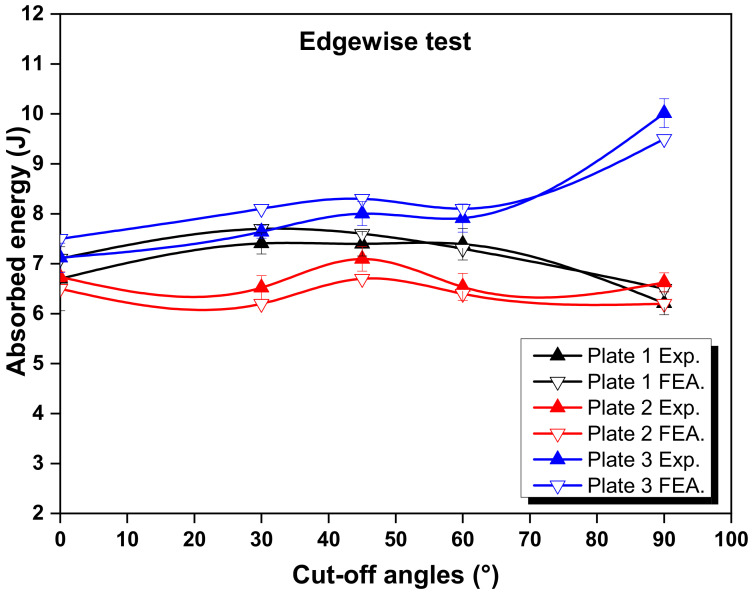
Absorbed energy at various cut-off angles for the edgewise test vs. finite element modelling.

Figure 3 and Figure 4 show the experimental and numerical results for the absorbed energy of the selected configurations from the flatwise and edgewise tests, respectively. It can be observed that higher values of absorbed energy were produced by Plate 3 compared to the conventional laminate (Plate 1) and Plate 2. Nevertheless, it can also be noticed that the laminate with a conventional ply thickness exhibited a higher amount of energy than Plate 2. Under the flatwise condition, the maximum experimental value was recorded for the configuration of Plate 3, which was around 8.7 J for the cut-off angle of 90°. This configuration showed a 22.5% and 27.1% higher energy absorption capacity than the configurations of Plates 1 and 2, respectively. This increment was due to effective load bearing and microcrack suppression, which prevented further cleavage [33]. The mixed configurations reduced the degree of cracking in the load-acting direction and provided the highest strength. For the edgewise impact, the maximum value was also observed for the configuration of Plate 3, which was around 10.0 J for the cut-off angle of 90°. This configuration showed 61.2% and 51.1% higher results than the configurations of Plates 1 and 2, respectively. In addition, an agreement was found between the experimental and finite element analysis results in terms of the higher absorbed energy values. Similar results were also reported by Saleh et al. [34] and Zachariah et al. [25], respectively. Those authors concluded that the fibre orientation and stacking order highly influence energy absorption at the point of impact.

It can be observed from Figure 3 and Figure 4, which refer to the experimental and finite element analysis (FEA) results, that the values of absorbed energy from the FEA were higher than the experimental values in most cases. The values of absorbed energy under flatwise conditions were also generally higher. This could be attributed to the contact surface area during the impact tests, which was more prominent in the flatwise cases. However, the configuration of Plate 3 showed different trends, particularly at a cut-off angle of 90°. This implied that the value of absorbed energy was higher in the edgewise cases. Consequently, the fibre directions played a very important role in carrying the load in the longitudinal direction, which resulted in a higher impact resistance. To explore this more deeply, the cut-off angles for each case were analysed. In the flatwise case for the configuration of Plate 1, the absorbed energy increased with the increase in angle, up to the cut-off angle of 45°. The configuration of Plate 2 showed the same trend. However, the maximum experimental and numerical absorbed energy values were found at the angle of 90° for Plate 3. For the edgewise cases, a similar pattern was observed to that of the flatwise results. The cut-off angle of 45° showed that the absorbed energy for both cases was somewhat similar. This was due to the load carrying fibres being perpendicular to the applied load [35]. The error ratio of the numerical results was minor compared to that of the experimental results for the flatwise and edgewise impacts. However, the variation was higher in Plate 3. Note that the maximum difference between the experimental and finite element simulation results did not exceed 9%, which demonstrated the validity of the proposed model.

The experimental and numerical results of the Charpy impact tests for various cut-off angles revealed that the effects of adding thin plies into the mid-plane of the proposed traditional laminate design were negligible in terms of energy absorption. This could be attributed to the damage that was initiated in the traditional plies reducing the stiffness at points of increased deformation, which started cracking in the thin plies within the damage-contacted areas [36]. During bending deformation, however, the arrangement of two thin plies surrounding each conventional ply in Plate 3 absorbed the most impact energy. This finding demonstrated that the use of thin plies as a cover for standard plies significantly delayed fibre failure. However, in order to interpret this behaviour, damage assessments also needed to be investigated. Due to the hybrid configuration of thin plies and traditional plies, matrix failure and interlaminar failure took place in between the plies, which affected the absorption of energy [37]. The absorbed energy during the Charpy impact tests was principally influenced by the fracture behaviour, matrix failure and delamination mechanisms. Furthermore, the cut-off angles of the laminates had a substantial impact on the absorbed energy values, both experimentally and numerically.

### 3.2. Damage Assessment

Table 3, Table 4 and Table 5 illustrate the outcomes of the damaged specimens at different cut-off angles for Plates 1, 2 and 3, respectively. The compensated impact strength of the Plate 3 Charpy specimen was much higher than that of specimens of Plates 1 and 2. Three types of failure behaviour were observed in the specimens of Plates 1, 2 and 3. The first type of failure occurred due to the breakage of fibres and the cracking of the ply matrix during impact loading. Second, there was delamination damage, which started at the impact point. For all configurations, normal delamination formed in the middle of the continuous damage region of the traditional plies, which caused the delamination damage. The stiffness of the traditional plies reduced as the damage progressed, which resulted in more deformation and caused the thin plies to break, thereby generating discontinuities in the estimated damage regions. The discontinuity in the damage areas indicated that the delamination formed at separate interfaces and was most likely unconnected [38]. Similar results were reported by Mencattelli et al. [39]. In that research study, the authors prepared a Herringbone–Bouligand laminate to improve resistance to impact and damage. According to their conclusions, the Herringbone–Bouligand laminate significantly reduced delamination damage (71%). Thus, it is clear that the fibre orientation and stacking order highly influence the damage resistance of high-performance fibre laminate composites. Moreover, interlaminar failure that occurred due to the weak interfacial bonding between the different configurations of plies and polymer matrices also impacted the absorption of energy [40]. The fibre and matrix composite specimens showed failure due to the concentration of shear stress at the damaged area, which varied according to the different cut-off angles [41]. This particular damage mechanism that is due to poor bonding effects was also reported by Prakash et al. [42]. Those authors concluded that the lack of adhesion behaviour results in poor load bearing effects in composites. It was noted that Plate 3 was highly resistant to the failures compared to Plates 1 and 2. The optimised configuration of the composite plate design improved the interlaminar fracture toughness and, consequently, increased the energy absorption capability. The obtained results indicated that the optimised configuration of traditional ply and thin ply improved the absorption of energy in the hybrid laminate, which is crucial for application purposes.

### 3.3. Validity of FEM Results

The FEA was established by comparing the test results and the modelling of absorbed energy at the various cut-off angles for all of the selected plates, as shown in Figure 3 and Figure 4. The correlations revealed a general trend of good agreement between the test results and the simulation findings. The failure modes of the hybrid laminates that used both thin and normal plies, which were tested with the Charpy impact test, could be summarised as fibre breakage, local indentation, matrix cracks, delamination and skin debonding at the impact point of the striker. The low-velocity Charpy impact test results were strongly dependent on the configuration, fibre and matrix type, the thickness of the ply, loading velocity and the variety of the projectile [43,44].

The simulation results are shown in Table 3, Table 4 and Table 5 and refer to the Plate 1 laminate (reference), the Plate 2 hybrid laminate and the Plate 3 hybrid laminate, respectively. When the hammerhead struck the plate, the stress was mostly localised at the fracture and the contact area, which indicated that when the plate deformed, the stress was concentrated on the crack and made it easier to break. With the continuous loading of the hammerhead velocity field, the fractured areas continued to expand and the stress was mainly concentrated near the fractured area. The damage shape results of the simulation process were partly consistent with the actual experimental results. However, some variations were observed due to the complex damage modes, which resulted from the high-impact energy that affected the specimen shapes. When comparing the experimental and finite element analysis results for Plate 1 in the flatwise impact direction (Table 3), it was found that delamination failure (layer separation), fibre and matrix cracks and debonding were the most common failures. This was attributed to the normal ply stacking sequences and thickness orientations. In the case of the edgewise direction, the striker impacted the thickness of the laminate because the consistency was slightly different from the other plates and the contact surface was very thin, which could change the shape of the damage that occurred after impact. The size of the damaged area was larger for the 0° and 30° cut-off angles than for the other cut-off angles. Despite the fact that the shape of the damage followed the same pattern as the experiments, the size of the damaged area was different. This was attributed to many factors, such as the mesh size and optimised loading times during the simulation process [45].

The damage that was formed on Plate 2 for the flatwise impact position (Table 4) showed the same pattern at the 0°, 30° and 60° cut-off angles, while the damage mode revealed delamination between the lower layers at other cut-off angles. Fibre breakage, matrix cracking and fibre debonding were the most common failures for Plate 2. The edgewise impact position of Plate 2 showed a good agreement between the experimental and finite element analysis results in terms of the damage shape. All of the damage shapes showed the same trend at all cut-off angles, which consisted of fibre breakage and matrix cracking. The comparison between the experimental and finite element analysis results for Plate 3 (Table 5) revealed delamination failure mode at the 0°, 45° and 90° cut-off angles. Fibre debonding and matrix cracking with indentations at the impact location were shown for the 30° and 60° cut-off angles; however, for the edgewise impact position, the damage shapes were different from the other plates.

As a comparison between all of the plates, a considerable delamination mode failure from the flatwise impact position occurred on Plate 1 at all cut-off angles. In addition, massive fibre breakage and debonding were observed in Plate 2 in order to consume the impact energy that resulted from the strong block in the middle. Although the FEM was capable of predicting the absorbed energy for all of the different configurations with good levels of accuracy, the prediction of complex damage modes could be improved by considering intra and interlaminar damage. However, the existing model could optimise the laminate design of different configurations for many applications.

## 4. Conclusions

The present study aimed to examine hybrid (thin and thick plies) composite laminate configurations under Charpy impact loading conditions. A conventional ply configuration and two hybrid configurations were designed and tested, both experimentally and numerically. The hybrid configurations of composite laminate were made with both thin plies and normal plies. The impact properties of the selected configurations were investigated using low-velocity Charpy impact tests and the effects of the cut-off angle were studied in the flatwise and edgewise directions. According to the test results, the following conclusions were made:

(i) The absorbed impact energy was significantly influenced by the hybridisation and the hybrid plies did not show any delamination effects due to poor adhesion;
(ii) The integration of thin plies that were made from fabric–epoxy composites in between each traditional ply increased the absorbed energy by up to 10 J for the edgewise configuration at a cut-off angle of 90°, whereas the flatwise energy absorption was recorded as 8.7 J;
(iii) For Plates 1 and 2, the cut-off angle of 45° seemed to produce higher energy absorption. However, for the Plate 3 configuration, a cut-off angle of 90° outperformed the others in terms of energy absorption;
(iv) The damage assessments showed the increased damage prevention of the hybrid thin ply composites because they distributed the stress more uniformly, whereas the traditional ply composites incurred large deformations from the impact loads;
(v) The FEM model was capable of accurately predicting the absorbed energy for the different configurations of composites that were prepared and analysed. Both experimental and numerical values were very similar;
(vi) The proposed hybrid composite laminates could be useful due to their improved impact energy and the existing models. However, the ply configuration could be optimised for other potential applications wherever possible.

## Figures and Tables

**Table 1:** Description of the properties of the CF–epoxy laminate that was used.

No.	Property	Unit	Value
1	Strength of fibre (σf)	GPa	3.1–3.3
2	Strength of matrix (σm)	MPa	60–66
3	Density of fibre (ρf)	g/cm^3^	1.75–1.93
4	Density of matrix (ρm)	g/cm^3^	1.18–1.20
5	Longitudinal stiffness (E_11_)	GPa	73–75
6	Transverse stiffness (E_22_)	GPa	7.733–7.92
7	Out-of-plane stiffness (E_33_)	GPa	7.733–7.76
8	Poisson’s ratio (ν_11_)		0.1
9	Poisson’s ratio (ν_13_)		0.1
10	Poisson’s ratio (ν_23_)		0.3
11	Shear modulus (G_12_)	GPa	4.3–4.5
12	Shear modulus (G_13_)	GPa	4.3–4.5
13	Shear modulus (G_23_)	GPa	2.3–2.4
14	Ply thickness	mm	0.085–0.33
15	Density of composite (ρ)	(kg/m^3^)	1840

**Table 2:** Description of the properties of the cohesive elements.

Property	Value
Longitudinal stiffness, *E* (MPa)	1.25 × 10^6^
Transverse stiffness, *G*_1_ (MPa)	1.25 × 10^6^
Out-of-plane stiffness, *G*_2_ (MPa)	1.25 × 10^6^
Nominal stress normal-only mode, *N*_0_ (MPa)	71
Nominal stress first direction, *T*_0_ (MPa)	88
Nominal stress second direction, *S*_0_ (MPa)	88
Normal mode fracture energy, *G*_1c_ (N/mm)	0.095
Shear model fracture energy first direction, *G*_2c_ (N/mm)	0.686
Shear model fracture energy second direction, *G*_3c_ (N/mm)	0.686
Power, *η*	1.45

**Table 3:** Failure modes of Plate 1 under Charpy impact test conditions.

Plate 1					
**Cut-Off Angle**	**EXP. Flatwise Failure**	**FEA. Flatwise Failure**	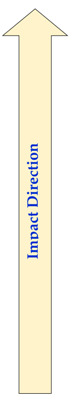	**EXP. Edgewise Failure**	**FEA. Edgewise Failure**
0	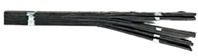	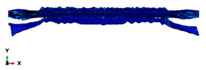		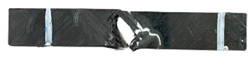	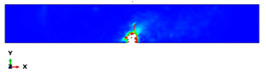
30	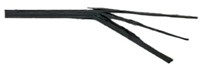	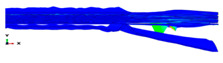		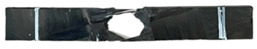	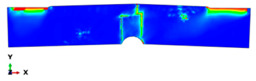
45	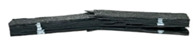	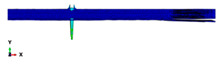		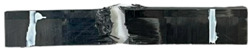	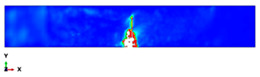
60	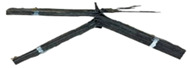	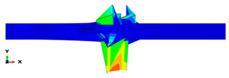		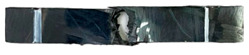	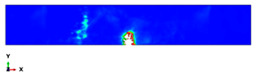
90	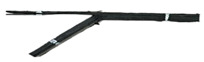	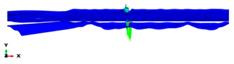		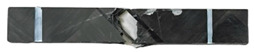	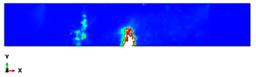

**Table 4:** Failure modes of Plate 2 under Charpy impact test conditions.

Plate 2					
**Cut-Off Angle**	**EXP. Flatwise Failure**	**FEA. Flatwise Failure**	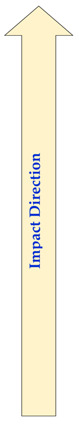	**EXP. Edgewise Failure**	**FEA. Edgewise Failure**
0	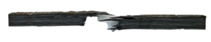	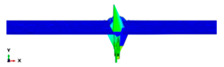		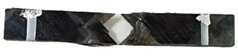	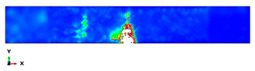
30	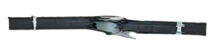	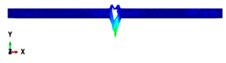		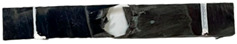	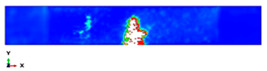
45	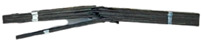	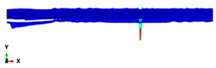		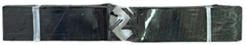	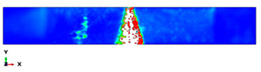
60	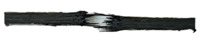	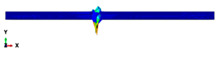		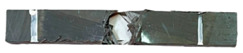	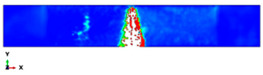
90	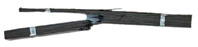	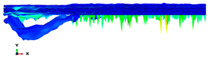		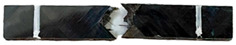	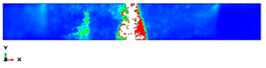

**Table 5:** Failure modes of Plate 3 under Charpy impact test conditions.

Plate 3					
**Cut-Off Angle**	**EXP. Flatwise Failure**	**FEA. Flatwise Failure**	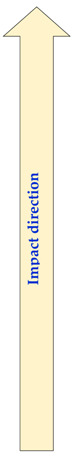	**EXP. Edgewise Failure**	**FEA. Edgewise Failure**
0	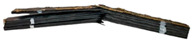	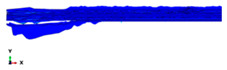		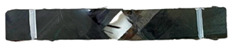	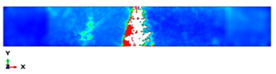
30	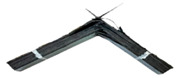	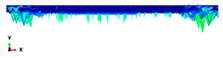		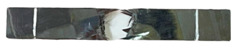	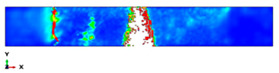
45	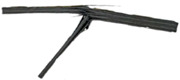	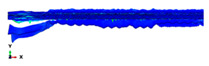		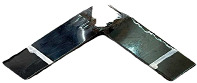	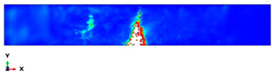
60	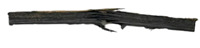	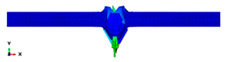		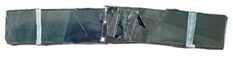	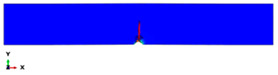
90	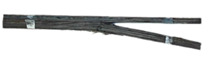	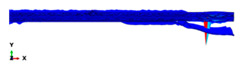		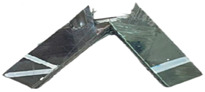	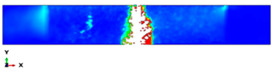

## Data Availability

All data are contained within the article.
